# Prevalence and Prognostic Value of Mediastinal Lymph Node Dissection in Pulmonary Metastasectomy: A Retrospective Single‐Center Analysis

**DOI:** 10.1111/1759-7714.70278

**Published:** 2026-04-02

**Authors:** Christian Galata, Jonathan Schulz, Laura Grifone, Sergio A. Zapata Bonila, Thomas Kindler, Kalliopi Athanassiadi, Eric Roessner, Ioannis Karampinis

**Affiliations:** ^1^ Department of Thoracic Surgery University Medical Center Mainz, Johannes Gutenberg University Mainz Mainz Germany; ^2^ University Cancer Center Mainz (UCT Mainz), University Medical Center Mainz, Johannes Gutenberg University Mainz Mainz Germany; ^3^ 3rd Medical Department University Medical Center Mainz, Johannes Gutenberg University Mainz Mainz Germany; ^4^ TRON‐Translational Oncology, University Medical Center, Johannes Gutenberg University Mainz Mainz Germany; ^5^ German Cancer Consortium (DKTK), partner Site Frankfurt/Mainz, a Partnership Between DKFZ and University Medical Center Mainz Mainz Germany; ^6^ Division of Thoracic Surgery Evaggelismos General Hospital Athens Greece

**Keywords:** colorectal metastasis, lung metastasectomy, lung metastasis, sarcoma metastasis

## Abstract

**Background:**

Pulmonary metastasectomy is an established part of the multimodal treatment of various malignant diseases. While the procedure typically focuses on complete removal of metastatic lesions, the role of mediastinal lymph node involvement remains unclear. We aimed to analyze the prevalence of lymph node involvement and its impact on prognosis in patients undergoing pulmonary metastasectomy.

**Methods:**

Patients with different primaries with histologically confirmed pulmonary metastases that underwent pulmonary metastasectomy with the goal of complete resection were included in this retrospective analysis. The type of lymph node dissection, the type of lung resection, the number of lymph nodes harvested as well as survival data were analyzed.

**Results:**

Among 219 patients, lymph node samples were obtained in 91 (41.6%). Lymph node metastases were identified in 13 patients (14.3%). No specific primary tumor entity showed a significantly higher risk of lymph node involvement. After a median follow‐up of 10 months, 12 of 13 lymph node positive patients (92.3%) had either radiologically confirmed active disease or had died of malignancy, compared with 47.1% in the lymph node negative group (*p* = 0.013).

**Conclusions:**

Mediastinal lymph node involvement in pulmonary metastasectomy is associated with poor prognosis and may occur across a broad spectrum of primary tumor entities. Our data may support the routine use of modern staging modalities such as PET/CT and EBUS in candidates for pulmonary metastasectomy, in order to better stratify patients and avoid unnecessary surgical interventions.

## Graphical abstract

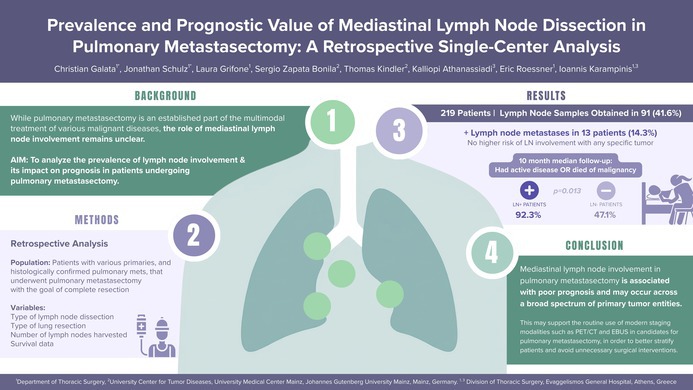

## 1 Introduction

Systematic mediastinal lymph node dissection is standard practice in lung cancer surgery where nodal status directly impacts prognosis and the definition of complete resection [1, 2]. The preoperative staging of the mediastinum is a complex, often time‐consuming process involving CT scan, PET scan, endobronchial ultrasound with fine‐needle aspiration (EBUS), esophageal ultrasound, surgical procedures like video‐assisted mediastinoscopy or even thoracoscopy, as well as combinations of these modalities [3].

The number of patients undergoing pulmonary metastasectomy has increased steadily during the last decade and is expected to increase even more in the future [4]. Appropriate patient selection remains challenging due to the heterogeneity of primary tumors, systemic treatment histories, and tumor biology. Several criteria have been proposed for selecting the right candidates for metastasectomy, including a low number of metastases, expected complete resection as well as the time interval between diagnosis of primary and treatment of metastatic disease [5, 6].

Several retrospective studies have pointed out the impact of mediastinal lymph node involvement in pulmonary metastasectomy. A systematic review of these studies concluded that the overall incidence of lymph node involvement is probably significantly underestimated and that these patients should be excluded from surgery of the metastatic disease [7]. An older cadaveral study reported an incidence of lymph node involvement reaching 33% in patients with extrapulmonary primaries [8]. A more recently published report by the European Society of Thoracic Surgery analyzed a big registry of patients undergoing pulmonary metastasectomy and concluded that lymph node biopsies (sampling or dissection) are performed in 58% of the population and that the role of lymph node assessment needs to be further studied [7, 9]. An American consensus report, on the other hand, suggests mediastinal lymph node sampling/dissection due to the impact on survival [6, 10].

The prognostic significance of lymph node involvement, combined with advances in non‐invasive staging modalities, raises the question of whether systematic lymph node staging should be implemented in metastasectomy candidates. In this retrospective single‐center study, we aimed to evaluate the prevalence of lymph node involvement in pulmonary metastasectomy and assess its prognostic implications.

## 2 Materials and Methods

The study was conducted in accordance with the Declaration of Helsinki and was approved by the local ethics committee (2021–15 979). Patients were included in this retrospective analysis over a period of 40 months. All patients admitted to our institution are requested to sign a consent for the use of their data for research purposes. Due to the retrospective nature of the study, no additional consent was required.

All patients that underwent pulmonary metastasectomy in our institution between January 2020 and April 2024 were included in this retrospective analysis. In all the presented cases, the purpose of the pulmonary metastasectomy was the oncological treatment of the disease with the intention to improve survival or potentially cure the disease. Data regarding the diagnosis of the primary, previous surgical procedures, previous pulmonary metastasectomies, number of resected metastases, type of the surgical procedure, and follow‐up data were extracted from the electronic medical record. Patients with missing data or patients that were not treated in our institution after surgery were excluded. Pediatric patients (< 16 years of age) were excluded as well.

The indication for pulmonary metastasectomy was discussed in disease specific multidisciplinary tumor boards, comprising radiologists, medical and radiation oncologists, pathologists and surgeons. The oncological criteria to perform pulmonary metastasectomy included complete surgical resectability of all metastases as well as stable disease for at least 3 months without treatment in two consecutive CT scans (1 mm slice thickness or less than 1 mm) of the chest. In case of coexisting extrapulmonary metastases or bilateral pulmonary metastases regular CT scans were performed to ensure stability and resectability of the coexisting lesions. Preoperative staging of the mediastinum (PET/CT or endobronchial ultrasound) was not routinely performed. An EBUS was only performed if the patient had pathological mediastinal lymph nodes on the CT scan. Patients with positive mediastinum were excluded from this study.

The preferred surgical approach was video‐assisted thoracoscopy, when complete surgical resection was expected and the number of metastases allowed thoracoscopic resection. The indication to perform an anatomic lung resection was solely based on the location of the lesion and the possibility to achieve negative margins. Furthermore, anatomic lung resections were performed in case of suspicion of lung cancer that had not been histologically confirmed before surgery. Intraoperatively, the resection was considered complete if the lesion was removed entirely, not in fragments, and was entirely covered by lung tissue. For over three metastases, the preferred approach was thoracotomy. Lymph node assessment was implemented gradually in our department following the publication of the consensus paper of the Society of Thoracic Surgeons. The decision to perform lymph node biopsies, sampling, or systematic nodal dissection was left at the discretion of the surgeon. Patients that underwent resection for potential lung cancer underwent systematic nodal dissection.

### 2.1 Data Analysis

Quantitative variables are presented as median with interquartile range (IQR). Bivariate analysis of categorical variables was performed using two‐tailed χ^2^ or the Fisher's exact test. 5% was used as a cut‐off for statistical significance. The statistical analysis was performed using SPSS Statistics software (version 29.0, IBM, Armonk, NY, USA).

## 3 Results

Two hundred eighty‐five patients underwent pulmonary metastasectomy with the intention to treat the underlying malignancy between January 2020 and April 2024. In 20 cases, the resected lesions proved to be benign, and the patients were excluded from the analysis. In another eight cases, we discovered a primary lung cancer, and in one case, a metastasis from a previously unknown colorectal cancer. These patients were excluded from the analysis as well. Overall, 219 patients were included.

The procedure was uneventful in 201 cases. In 18 cases, the patients developed postoperative complications, three of which required intervention or surgery (new chest drain due to hemothorax and chylothorax respectively and surgery in another case due to postoperative bleeding).

The cohort comprised patients with the following primary tumor types: malignant melanoma (*n* = 15), head and neck cancer (*n* = 22), gastrointestinal malignancies (*n* = 59), gynecological malignancies (*n* = 9), hepatobiliary malignancies (*n* = 10), sarcoma (*n* = 59), genitourinary malignancies (*n* = 27); the remaining patients had other primary tumors. The time interval between diagnosis of the primary and diagnosis of metastatic disease was 16 months (IQR 31). One hundred sixteen patients had undergone previous metastasectomy, including 75 patients with a prior pulmonary metastasectomy. Eighteen patients had coexisting extrapulmonary metastases at the time of the lung metastasectomy. In 50 of 219 patients, up to 3 metastases were resected, whereas in the remaining 169 cases, patients had more than 3 metastases resected. The number of postoperative complications was significantly higher in the group of patients, where more than 3 metastatic lesions were resected (*p* = 0.006).

A thoracoscopic approach was chosen in 113 procedures (51.6%). The median number of resected lung metastases was 2 (IQR 0, range 1–88). Sixty‐nine patients underwent an anatomic lung resection (segmentectomy or lobectomy) for the resection of the pulmonary metastases. The patient characteristics are summarized on Table 1.

**TABLE 1:** Patient characteristics.

	Neg. LN (*n* = 78)	Pos. LN (*n* = 13)	*p*
Age > 70	15	2	0.31
Prev. metastasect.	43	8	0.76
Number of met. > 3	22	3	1.00
Extrapulm. met.	22	7	0.10
VATS	26	3	0.53
Anatomic resections	53	7	0.35
Postoperative treatment			
No treatment	32	2	
Systemic treatment	35	7	
Chemoradiotherapy	2	1	
Radiotherapy	5	2	
Unknown	3	1	

Abbreviations: met.: metastases; Neg. LN: negative lymphnodes; Pos. LN: positive lymphnodes; VATS: video assisted thoracoscopic surgery.

### 3.1 Lymph Node Involvement

In 91 cases lymph node samples were obtained during the metastasectomy procedure. Sixty‐three of 91 patients underwent a formal systematic nodal dissection and 28 patients underwent lymph node sampling. A median of five lymph nodes were harvested in total in the 91 cases (IQR 6, range 1–36). Lymph nodes were affected in 13 patients (14.3%). Of the 13 patients with lymph node involvement, 5 had gastrointestinal tumors, 1 prostate cancer, 2 malignant melanoma, 1 breast cancer, 1 head and neck cancer, 2 renal cancer and 1 sarcoma. Six of 13 patients had undergone a systematic lymph node dissection and 7 lymph node sampling. Three of the 13 patients were operated thoracoscopically and the other 10 through an anterolateral thoracotomy (Table 2). None of the patients that underwent lymph node sampling or dissection developed any complications.

**TABLE 2:** Patients with positive lymphnodes.

	Primary	Prev. metastasectomies	Surg. approach	Resected mets.	ALR
1	GI	Yes	VATS	**≤ 3**	No
2	Urogenital	No	Thoracotomy	**≤ 3**	Yes
3	Breast	No	Thoracotomy	**≤ 3**	No
4	GI	Yes	Thoracotomy	**≤ 3**	No
5	Urogenital	Yes	Thoracotomy	> 3	No
6	Sarcoma	Yes	Thoracotomy	**≤ 3**	Yes
7	Urogenital	Yes	Thoracotomy	**≤ 3**	Yes
8	GI	No	Thoracotomy	> 3	Yes
9	ENT	No	VATS	**≤ 3**	No
10	GI	Yes	Thoracotomy	**≤ 3**	Yes
11	Melanoma	No	Thoracotomy	> 3	No
12	GI	Yes	VATS	**≤ 3**	Yes
13	Melanoma	Yes	Thoracotomy	**≤ 3**	yes

Abbreviations: ALR: anatomic lung resection; ENT: ear‐nose‐throat; GI: gastrointestinal; Mets.: metastases; Prev.: previous.

In order to analyze if the extent of lymph node dissection is associated with the incidence, we divided the patients into a group that underwent systematic nodal dissection and a group that underwent sampling. In the 63 patients that underwent formal mediastinal lymph node dissection, 6 had affected lymph nodes (9.5%) compared with 7 patients in the group that underwent sampling (25%). Furthermore, we analyzed if the surgical approach was associated with the outcome. The 29 patients that were operated on thoracoscopically were compared with the 62 patients that underwent thoracotomy. There was no difference in the number of patients that were alive, with or without disease, at the end of the follow‐up period (*p* = 0.77).

### 3.2 Follow Up Data

Thirty eight patients received no further therapy immediately after the pulmonary metastasectomy. The remaining 181 received systemic treatment, radiotherapy or combinations of the two. The interval between the pulmonary metastasectomy and the follow‐up was 10 months (IQR 16). Two hundred one patients were alive at follow‐up, 74 of which were without evidence of active disease. Of the 13 patients that had lymph node involvement, only one was alive and tumor‐free at follow‐up, while 10 patients were alive with disease and two had passed away due to the malignant disease. Of the 78 patients without lymph node metastases, 70 were alive at follow‐up and 37 were alive without evidence of disease (52.9%). Active disease at follow‐up was significantly more common in the group of patients with lymph node metastases than in the patients where the lymph nodes were not involved (*p* = 0.013).

## 4 Discussion

Proper patient selection is critical in pulmonary metastasectomy. The presence of lymph node metastases has been described as a negative prognostic factor. However, the mediastinum is not routinely staged with PET/CT or EBUS in these patients. This contrasts with lung cancer pathways, where PET/CT and EBUS are standard.

Following the recommendations of the Society of Thoracic Surgery, we started implementing lymph node biopsies as a standard in surgery of metastatic disease at our institution [6]. In this study, we retrospectively evaluated the outcomes of patients undergoing pulmonary metastasectomy and investigated the impact of lymph node involvement in these cases.

Our study confirms that mediastinal lymph node involvement in pulmonary metastasectomy is not uncommon (approx. 14%). Almost all these patients had either active disease or had died of the malignant disease within 10 months from surgery. Importantly, lymph node metastases were detected across a broad range of primary tumors, emphasizing that a wide range of malignancies can have mediastinal involvement. Our findings are consistent with prior retrospective series and registry data, which report lymph node involvement in 10%–30% of cases and a significant survival disadvantage [11, 12, 13, 14, 15].

Surgery leads to oxidative stress, affects the overall condition of the patient, and might interfere with tumor growth [16, 17]. Furthermore, surgery can cause delays to systemic therapy, which may especially disadvantage lymph node positive patients. That makes the selection process for the proper candidates for surgical metastasectomy critical.

Positive mediastinal lymph nodes are a strong prognostic factor for poor prognosis of pulmonary metastasectomy. The results of our study support the idea of optimizing the selection process of patients with pulmonary metastases in order to narrow down the surgical candidates to those with no lymph node involvement and potentially a more favorable prognosis. Intensified preoperative staging may prevent patients with mediastinal metastases from undergoing procedures with limited oncological benefit and unnecessary morbidity.

### 4.1 Limitations

The collection of the data in this study was performed prospectively but the interpretation and processing were performed retrospectively and are therefore subject to all sorts of bias that retrospective studies are susceptible to. Furthermore, the preoperative staging/mediastinal staging was inconsistent among the patients included and the indication to perform any sort of nodal assessment was surgeon‐based and not standardized. However, despite the retrospective nature of the study, all relevant information was available. Furthermore, this is a large cohort of patients with different tumor entities, different preceding treatments, staging, tumor load, and so forth, which makes the sample heterogeneous and therefore limits the generalizability and strength of the conclusions.

## 5 Conclusion

Lymph node involvement in pulmonary metastasectomy occurs in around 14% of the patients and is associated with poor prognosis. Properly staging the mediastinum in patients referred for pulmonary metastasectomy might be the right approach in order to narrow down these patients to the ones that will probably profit from the procedures and preclude patients with involved mediastinum from getting involved with surgery and delaying more vital treatment options.

## Author Contributions

C.G. conceived of the presented idea, collected, and analyzed the data. J.S. collected and analyzed the data. L.G. collected and analyzed the data. S.A.Z.B. collected and analyzed the data. T.K. has reviewed the manuscript for important intellectual content. K.A. has reviewed the manuscript for important intellectual content. E.R. has reviewed the manuscript for important intellectual content. I.K. conceived of the presented idea, collected and analyzed the data, and drafted the manuscript.

## Funding

The authors have nothing to report.

## Ethics Statement

The study was conducted in accordance with the Declaration of Helskinki and was approved by the local ethics committee (2021‐15 979).

## Consent

Due to the retrospective nature of the study, no written informed consent was obtained.

## Conflicts of Interest

The authors declare no conflicts of interest.

## Data Availability

The datasets generated during the current study are not publicly available since the main part is included in this article. The complete database is available from the corresponding author on reasonable request.
